# Alternating Direction Method of Multipliers-Based Constant Modulus Waveform Design for Dual-Function Radar-Communication Systems

**DOI:** 10.3390/e25071027

**Published:** 2023-07-06

**Authors:** Ahmed Saleem, Abdul Basit, Muhammad Fahad Munir, Athar Waseem, Wasim Khan, Aqdas Naveed Malik, Salman A. AlQahtani, Amil Daraz, Pranavkumar Pathak

**Affiliations:** 1 Department of Electrical & Computer Engineering, Faculty of Engineering & Technology, International Islamic University, Islamabad 44100, Pakistan; ahmed.phdee11@iiu.edu.pk (A.S.); abdulbasit@iiu.edu.pk (A.B.); fahad.munir@iiu.edu.pk (M.F.M.); athar.waseem@iiu.edu.pk (A.W.); wasim.khan@iiu.edu.pk (W.K.); anaveed@iiu.edu.pk (A.N.M.); 2 Department of Computer Engineering, College of Computer and Information Sciences, King Saud University, P.O. Box 51178, Riyadh 11543, Saudi Arabia; salmanq@ksu.edu.saa; 3 School of Information Science and Engineering, NingboTech University, Ningbo 315100, China; 4 School of Continuing Studies, McGill University, Montreal, QC H3A 0G4, Canada; pranavpp@gmail.com

**Keywords:** dual-function radar communication, MIMO communication, waveform design, ADMM

## Abstract

In this paper, we design constant modulus waveforms for dual-function radar-communication (DFRC) systems based on a multi-input multi-output (MIMO) configuration of sensors for a far-field scenario. At first, we formulate a non-convex optimization problem subject to waveform synthesis for minimizing the interference power while maintaining a constant modulus constraint. Next, we solve this non-convex problem, iteratively, using the alternating direction method of multipliers (ADMM) algorithm. Importantly, the designed waveforms approximate a desired beampattern in terms of a high-gain radar beam and a slightly high gain communication beam while maintaining a desired low sidelobe level. The designed waveforms ensure an improved detection probability and an improved bit error rate (BER) for radar and communications parts, respectively. Finally, we demonstrate the effectiveness of the proposed method through simulation results.

## 1. Introduction

The exponential growth of the wireless communication industry, producing billions of connected devices, has led to a severe problem of frequency spectrum congestion [1,2,3]. Unfortunately, new mobile network operators and emerging technologies are facing spectrum scarcity. Consequently, the auction prices of the wireless frequency spectra have risen sharply in recent years [4,5,6]. Therefore, different methods have been proposed to improve the coexistence between active sensing systems (i.e., spectrum sharing) [7,8,9,10,11]. On the other hand, the comprehensive studies have shown that the radar bands are mostly under-utilized and can be used for communication purposes. Therefore, the use of radar bands for communication purposes has increased in recent years. For example, the L-band (1–2 GHz) is shared by the long-range surveillance and air traffic control (ATC) radars with supported communication technologies, such as LTE and 5 G NR. Additionally, milli-meter wave (mmWave) bands (30–300 GHz) are shared by automotive and high-resolution imaging radars with supporting IEEE 802.11ad/ay and WLAN communication technologies. Similarly, S-band (2–4 GHz) and C-band (4–8 GHz) are also being shared for communication purposes. These spectrum sharing methods gave birth to a new technology named as joint radar-communication designs, which is also called communication-radar spectrum sharing (CRSS) [12], RadCom [13], or integrated sensing and communication (ISAC) [14,15] designs in a broader sense. The schemes, in which the radar and communication systems utilize the same frequency bands but exist as separate systems, fall in the general category of radar-communication coexistence (RCC) [16].

Besides spectrum scarcity, multifunction antenna sensors are another motivation for the joint radar-communication designs [17]. Because a multifunction antenna sensor integrates communication, radar, and electronic counter-measure (ECM) subsystems on a single platform using shared antenna sensor arrays, the new designs based on multifunction antenna sensors facilitate flexible, robust, and adaptive processing for meeting the need of the day [18]. Importantly, by combining the radar and communication subsystems, the size, production/maintenance costs, and energy requirements of the devices can be significantly decreased. These systems are named as dual-function radar-communication (DFRC) [19,20,21], integrated radar and communication system (IRCS) [22] designs, etc., in the literature [23]. Precisely, the integrated radar-communication systems are particularly relevant to autonomous car networks and flying ad hoc networks (FANETs), in which vehicles sense and communicate with each other in order to know the traffic environment while simultaneously exchanging information with other vehicles [24,25].

Because both the radar and communication technologies are based on the same foundations of antenna theory, the unification of radar and communication systems seems straightforward. However, the two systems have different requirements and their simultaneous operation poses serious challenges in the design of integrated waveforms. Therefore, the research community has focused special attention on the waveform design techniques in this regard. Different waveform design methods are used depending on the type of radar-communication scheme. In the coexistence scheme, the radar and communication waveforms have been designed independently such that mutual interference is minimized between the systems. A time-sharing scheme has been proposed in [26], in which both of the systems work in the same frequency bands but not simultaneously. A second scheme, based on spatio-spectral coexistence [27], involves radar and communication systems operating at different frequencies. However, this scheme reduces the communication data rate and the radar range resolution. There is an interference avoidance method based on null-space precoding [28,29,30]. This design, however, has an adverse effect on radar performance. In another approach, radar sub-sampling matrix optimization has been performed for coexistence between a multi-input multi-output (MIMO) communication system and MIMO matrix-completion (MIMO-MC) radar [31,32], which gives a non-optimal performance to balance a trade-off between radar and communication. Because the co-design approaches with integrated waveforms allow simultaneous radar and communication operations, these generally utilize orthogonal frequency division multiplexing (OFDM) technology [33,34,35,36], which already has wide applications in radar and communication. Additionally, pulsed OFDM waveforms have been used in [37,38], where each pulse consists of several OFDM symbols. Wen et al. considered the transmit waveform design problem via a hybrid linear–nonlinear precoding signaling scheme where the joint waveform was designed as a superposition of nonlinearly precoded waveform and linearly precoded communication symbols [39]. Moreover, in [40,41], the radar waveform is modified by embedding communication information. However, performance of one of the systems is compromised in each of the aforementioned cases. On the other hand, the DFRC schemes have been proposed in [42,43,44], which facilitate the weight vector design to generate the desired beampatterns. In [43], the mainlobes in the beampatterns have been kept constant for the radar, while different phases [42], different sidelobe levels, or both [44] are used to encode communication bits. However, the waveforms do not follow constant modulus constraints in such schemes.

An important consideration in the design of suitable waveforms is the requirement to maintain a constant modulus [45,46,47] to obtain an improved performance. Because the constant modulus waveforms ensure energy-efficient transmission by avoiding signal distortion in amplifiers [48], these are well-suited for the current era radar and communication-based equipment. However, inclusion of the constant modulus constraint (CMC) in the design leads to non-convex optimization problem formulations which are NP-hard to solve. Different methods have been proposed in the literature to deal with the non-convex CMC problem [49,50], e.g., semidefinite relaxation is used in [49] to design code radar waveforms and sequential optimization procedures are proposed in [50] to design constant modulus waveforms for MIMO radar. Additionally, a manifold-based algorithm has been proposed in [51] to solve the constant envelope precoding problem. Moreover, a strategy based on antenna selection is proposed in [52] for MIMO DFRC systems where several sparse antenna selection and permutation methods are used for embedding communication bits in the radar emissions. However, as full array sensors have not been used, emissions have wide mainlobes and high sidelobes problems. Additionally, different optimization methods have been proposed in [53] to decrease the downlink multi-user interference in communication operation and generate the desired radar beampatterns. However, because the waveform formulation in the radar direction is unconstrained, the radar detection performance deteriorates drastically. In [46], different methods have been presented for integrated waveforms through a waveform synthesis (WS) constraint. These methods synthesize the desired radar and communication waveforms in different directions; however, they give a high peak-to-average power ratio (PAPR) or high sidelobe levels.

In this paper, we investigate a constant modulus waveforms design to approximate a desired beampattern for dual-function radar-communication based on a MIMO system. Note that the desired beampattern consists of a high-gain radar main beam with a slightly high gain communication beam while maintaining the desired low sidelobe levels. First, we formulate the waveform design problem as an optimization problem. Because the constant modulus constraint makes the problem non-convex and NP-hard, traditional methods cannot be applied to solve the problem. Therefore, we use a well-known alternating direction method of multipliers (ADMM) algorithm to obtain an iterative solution to the problem. The ADMM blends the idea of the augmented Lagrangian method (ALM) with the dual decomposition method [54,55] to achieve an improved performance. The designed waveforms ensure an improved detection probability and bit error rate (BER) for radar and communications parts, respectively. Finally, the simulation results validate the efficiency of the proposed design in terms of convergence, approximation of the desired waveform, and beampattern synthesis.

This paper is organized as follows. Section 1 gave a brief introduction of the study field and a review of the relevant literature. The signal model is explained in Section 2. Section 3 explains how the waveform has been formulated mathematically as non-convex optimization. In Section 4, the design problem is manipulated such that ADMM can be applied to it. The simulation results are provided in Section 5. Finally, the discussion of this paper is summarized and concluded in Section 6.

Notations: The notations R and C, used in this paper, represent the real and complex sets, respectively, while ℜ(·) and ℑ(·) denote the real and imaginary parts of the argument, respectively. The notation ⊗ represents the Kronecker product, |·| represents the absolute value of the argument, ${\left\|\cdot \right\|}_{2}$ represents the $l_{2}$ norm, and $I_{N}$ represents the N×N identity matrix. Table 1 provides a list of the abbreviations used in this paper.

## 2. Signal Model

**Figure 1:**
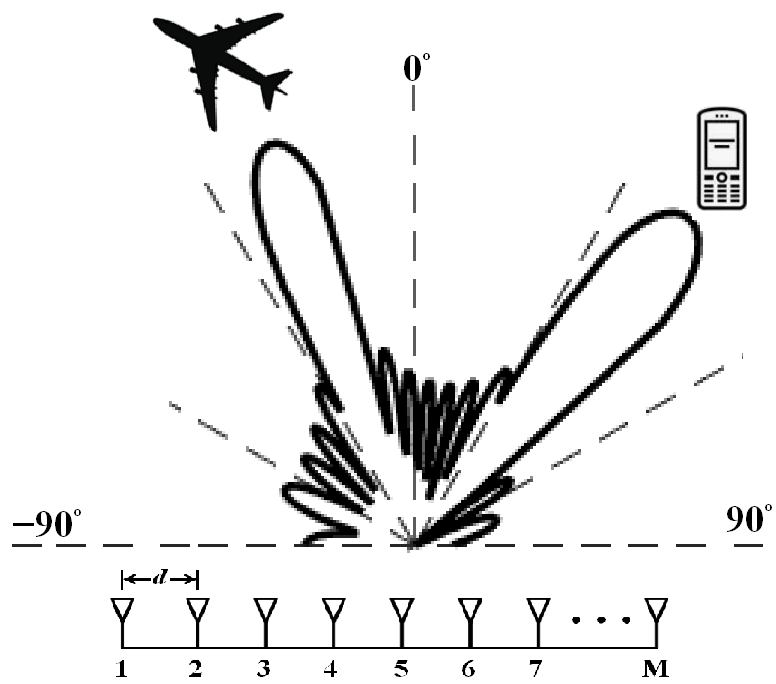
System model.

Consider a multi-input, multi-output joint radar-communication system, which is equipped with a uniform linear array (ULA), consisting of M transmit antenna sensors, as shown in Figure 1. Moreover, the radar and communication receivers consist of *M* transmit antenna elements. The antenna array transmits an integrated waveform for radar target detection that is also decoded at the communication end for detecting encoded information.

Let $s_{m}\left(n\right)$∈C denote the *n*th sample of a discrete waveform, consisting of *N* samples, emitted by the *m*th antenna, where m=1,…,M and n=1,…,N. Let s(n) denote a vector that collects the *n*th samples of the waveforms transmitted by all antennas, i.e., $s\left(n\right)={\left[s_{1}\left(n\right),\ldots ,s_{M}\left(n\right)\right]}^{T}$. Then, the far-field waveform in the direction θ is given by

(1) $$x\left(n;\theta \right)=a^{H}\left(\theta \right)s\left(n\right)$$

where

(2) $$a\left(\theta \right)={\left[1,e^{-j2\pi dsin(\theta )/\lambda },\ldots ,e^{-j2\pi (M-1)dsin(\theta )/\lambda }\right]}^{T}$$

is the transmit steering vector, with λ being the wavelength and *d* the inter-element spacing between the individual antenna elements. Let $S=[s_{1},\ldots ,s_{N}]$ be the M×N space-time transmit waveform matrix.

Let $x_{R}={\left[x_{R}\left(0\right),\ldots ,x_{R}\left(N-1\right)\right]}^{T}$ be the desired radar waveform and $x_{C}=[x_{C}\left(0\right),\ldots ,$ $x_{C}{\left(N-1\right)]}^{T}$ be the desired communication waveform. The transmit waveform matrix S is designed such that the $x_{R}$ is synthesized in radar direction $\theta_{R}$ and $x_{C}$ in communication direction $\theta_{C}$, respectively, where $\theta_{R}\neq \theta {}_{C}$, i.e.,

(3) $$a\left(\theta_{R}\right)S=x_{R}^{T}$$

and

(4) $$a\left(\theta_{C}\right)S=x_{C}^{T}.$$

Equations (3) and (4) can be combined as

(5) $$A^{H}\left(\Theta \right)S=X,$$

where $A\left(\Theta \right)=\left[a\left(\theta_{R}\right),a\left(\theta_{C}\right)\right]$ and $X={\left[x\left(\theta_{R}\right),x\left(\theta_{C}\right)\right]}^{T}$.

## 3. Problem Formulation

The problem under consideration is to design a transmit waveform matrix **S** so that the power radiation in the sidelobe region can be minimized. Moreover, there are two constraints. The first constraint is the waveform synthesis (WS) constraint, i.e., the transmit waveform matrix S synthesizes the desired radar waveform $x_{R}$ and the desired communication waveform $x_{C}$ in the direction of the radar target and communication users, respectively, as given by Equation (5). The second constraint is the constant modulus constraint which prevents the nonlinear signal distortion in the amplifiers to increase the efficiency of the transmitter. The constant modulus constraint is expressed as

(6) |S(m,n)|=1,0≤m≤M−1;0≤n≤N.

The problem can be formulated as an optimization problem given by

(7) $$\begin{array}{cccc} \; & \underset{S}{\mathrm{minimize}}\; & \; & \|A^{H}\left(\tilde{\Theta }\right){S\|}_{F}^{2}\\ \; & \mathrm{subject}\;\mathrm{to}\; & \; & A^{H}\left(\Theta \right)S=X\\ \; & \; & & |S(m,n)|=1,0\leq m\leq M-1;0\leq n\leq N, \end{array}$$

where $\Theta =\left[\theta_{R},\theta_{C}\right]$ is the collection of radar and communication direction angles while $\tilde{\Theta }=\left[\theta_{1},\theta_{2},\cdots ,\theta_{K}\right]$ is the collection of angles of *K* sidelobes.

The CM constraint Equation (6) renders the optimization problem (7) as non-convex. Being NP-hard, this problem is difficult to solve using any convex optimization methods. The problem (7) can be re-formulated for ease of analysis in two steps: the vectorization step and the realization step.

### 3.1. Vectorization

In the vectorization step, the matrices **S** and **X** are vectorized by stacking all of their respective column vectors into single column vectors. Correspondingly, matrices $A\left(\Theta \right)$ and $A\left(\tilde{\Theta }\right)$ are also updated. This is given by

(8) $$\begin{array}{cc} \; & \bar{s}=\mathrm{vec}\left(S\right)\\ \; & \bar{x}=\mathrm{vec}\left(X\right)\\ \; & \bar{A}\left(\Theta \right)=I_{N}⊗A\left(\Theta \right)\\ \; & \bar{A}\left(\tilde{\Theta }\right)=I_{N}⊗A\left(\tilde{\Theta }\right). \end{array}$$

The CM constraint is given in terms of $\bar{s}$ as

(9) $$\left|\bar{s}\left(i\right)\right|=1,i=1,2,\cdots ,MN$$

which is, equivalently, given by

(10) $${\bar{s}}^{T}E_{i}\bar{s}=1,i=1,2,\cdots ,MN$$

where

(11) $$E_{i}\left(m,n\right)=\left\{\begin{array}{cc} 1, & \;m=n=i\\ 0, & \;otherwise \end{array}\right.$$

where 0≤m,n,i≤MN. At the end of the vectorization step, the problem Equation (7) can be expressed as

(12) $$\begin{array}{cccc} \; & \underset{\bar{s}}{\mathrm{minimize}}\; & \; & {\bar{s}}^{H}\bar{A}\left(\tilde{\Theta }\right){\bar{A}}^{H}\left(\tilde{\Theta }\right)\bar{s}\\ \; & \mathrm{subject}\;\mathrm{to}\; & \; & {\bar{A}}^{H}\left(\Theta \right)\bar{s}=\bar{x}\\ \; & \; & & {\bar{s}}^{T}E_{i}\bar{s}=1,i=1,2,\cdots ,MN. \end{array}$$

### 3.2. Realization

In the realization step, the complex-valued variables are converted to the real-valued version. For example, the realization of $\bar{s}$ takes the real part of $\bar{s}$ in one column vector and the complex part in another vector and then stacks the column vectors together to give ${\bar{s}}_{r}$. The realization of ${\bar{s}}_{r}$, ${\bar{x}}_{r}$, ${\bar{A}}_{r}\left(\Theta \right)$, and ${\bar{A}}_{r}\left(\tilde{\Theta }\right)$ is given as

(13) $$\begin{array}{cc} \; & {\bar{s}}_{r}=\left[\begin{array}{c} ℜ\{\bar{s}\}\\ ℑ\{\bar{s}\} \end{array}\right]\\ \; & {\bar{x}}_{r}=\left[\begin{array}{c} ℜ\{\bar{x}\}\\ ℑ\{\bar{x}\} \end{array}\right]\\ \; & {\bar{A}}_{r}\left(\Theta \right)=\left[\begin{array}{cc} ℜ\{\bar{A}\left(\Theta \right)\} & -ℑ\{\bar{A}\left(\Theta \right)\}\\ ℑ\{\bar{A}\left(\Theta \right)\} & ℜ\{\bar{A}\left(\Theta \right)\} \end{array}\right]\\ \; & {\bar{A}}_{r}\left(\tilde{\Theta }\right)=\left[\begin{array}{cc} ℜ\{\bar{A}\left(\tilde{\Theta }\right)\} & -ℑ\{\bar{A}\left(\tilde{\Theta }\right)\}\\ ℑ\{\bar{A}\left(\tilde{\Theta }\right)\} & ℜ\{\bar{A}\left(\tilde{\Theta }\right)\} \end{array}\right] \end{array}$$

In terms of vectorized real-valued variables, the CM constraint is given by

(14) $${\bar{s}}_{r}^{T}E_{i}{\bar{s}}_{r}=1,i=1,2,\cdots ,2MN$$

where

(15) $$E_{i}\left(m,n\right)=\left\{\begin{array}{cc} 1 & :\;m=n=i\\ 1 & :\;m=n=i+MN\\ 0 & :\;otherwise \end{array}\right.$$

and 0≤m,n,i≤2MN. At the end of the realization step, the problem Equation (12) can be expressed as

(16) $$\begin{array}{cccc} \; & \underset{{\bar{s}}_{r}}{\mathrm{minimize}}\; & \; & {\bar{s}}_{r}^{T}{\bar{A}}_{r}\left(\tilde{\Theta }\right){\bar{A}}_{r}^{T}\left(\tilde{\Theta }\right)s_{r}\\ \; & \mathrm{subject}\;\mathrm{to}\; & \; & {\bar{A}}_{r}^{T}\left(\Theta \right){\bar{s}}_{r}={\bar{x}}_{r}\\ \; & \; & & {\bar{s}}_{r}^{T}E_{i}{\bar{s}}_{r}=1,i=1,2,\cdots ,MN. \end{array}$$

The optimization problem in Equation (16) can be solved to obtain ${\bar{s}}_{r-opt}$, which is the vectorized and real-valued version of $S_{opt}$. So, the reverse operation, i.e.,

(17) $$S_{opt}=mtx\left({\bar{s}}_{r1-opt}+i\cdot {\bar{s}}_{r2-opt}\right)$$

can be performed to obtain $S_{opt}$, where ${\bar{s}}_{r1-opt}$ contains the first MN elements, the real part, and ${\bar{s}}_{r2-opt}$ contains the other MN elements, the imaginary part.

## 4. ADMM Formulation and Solution

The optimization problem Equation (16), like Equation (7), is non-convex and NP-hard. Analytical solutions to problems such as this are challenging to obtain and alternatives such as numerical or heuristic techniques are employed instead to obtain approximate solutions. Even using heuristic techniques, such as a genetic algorithm (GA), it may be difficult to formulate the CM constraint. Therefore, we use the ADMM-based iterative technique to approximate a solution to this problem.

An auxiliary variable ${\bar{r}}_{r}$ is introduced in Equation (16) and the following equivalent version is obtained:

(18) $$\begin{array}{cccc} \; & \underset{{\bar{r}}_{r},{\bar{s}}_{r}}{\mathrm{minimize}}\; & \; & {\bar{r}}_{r}^{T}A_{r}\left(\tilde{\Theta }\right)A_{r}^{T}\left(\tilde{\Theta }\right){\bar{s}}_{r}\\ \; & \mathrm{subject}\;\mathrm{to}\; & \; & A_{r}^{T}\left(\Theta \right){\bar{r}}_{r}+A_{r}^{T}\left(\Theta \right){\bar{s}}_{r}=2{\bar{x}}_{r}\\ \; & \; & & T\left({\bar{r}}_{r}\right){\bar{s}}_{r}-1=0\\ \; & \; & & \bar{r}=\bar{s}. \end{array}$$

It can be observed that for the WS constraint in Equation (16), ${\bar{s}}_{r}$ is expressed as two times ${\bar{s}}_{r}$ and one of them is replaced by ${\bar{r}}_{r}$ in Equation (18). Moreover, the CM constraint, consisting of MN equations in Equation (16), is expressed in its compact form in (18) as

(19) $$G\left({\bar{r}}_{r},{\bar{s}}_{r}\right)=G\left({\bar{s}}_{r},{\bar{r}}_{r}\right)=0$$

where $G\left({\bar{r}}_{r},{\bar{s}}_{r}\right)\in R^{2MN\times 2MN}$ is a vector given by

(20) $$G\left({\bar{r}}_{r},{\bar{s}}_{r}\right)=T\left({\bar{r}}_{r}\right){\bar{s}}_{r}-1,$$

where 1 and 0 are 2MN×1 vectors, all 1s and 0s, respectively, and

(21) $$T\left({\bar{r}}_{r}\right)=\left[{\bar{r}}_{r}^{T}E_{1};{\bar{r}}_{r}^{T}E_{2};\cdots ;{\bar{r}}_{r}^{T}E_{MN}\right]\in R.^{MN\times 2MN}$$

The augmented Lagrangian of Equation (18) is given as

(22) $$\begin{array}{ccc} L\left\{{\bar{r}}_{r},{\bar{s}}_{r},u,v,w,\right\} & = & {\bar{r}}_{r}^{T}A_{r}\left(\tilde{\Theta }\right)A_{r}^{T}\left(\tilde{\Theta }\right){\bar{s}}_{r}\\ \; & + & \frac{\rho_{1}}{2}{∥A_{r}^{T}\left(\Theta \right){\bar{r}}_{r}+A_{r}^{T}\left(\Theta \right){\bar{s}}_{r}-2{\bar{x}}_{r}+u∥}_{\;2}^{\;\;2}\\ \; & + & \frac{\rho_{2}}{2}{∥T\left({\bar{r}}_{r}\right){\bar{s}}_{r}-1+v∥}_{2}^{2}\\ \; & + & \frac{\rho_{3}}{2}{∥{\bar{r}}_{r}-{\bar{s}}_{r}+w∥}_{2}^{2} \end{array}$$

where $u\in R^{4N\times 1},v\in R^{MN\times 1}$, and $w\in R^{2MN\times 1}$ are the dual variables and $\rho_{1},\rho_{2},\rho_{3}>0$ are the adjustable penalty parameters.

The (m+1)th iteration of the algorithm, in terms of the different variables, is given as follows:

(23a) $${\bar{r}}_{r}^{m+1}:=\underset{{\bar{r}}_{r}}{\arg\;\min}L\left({\bar{r}}_{r},{\bar{s}}_{r}^{m},u^{m},v^{m},w^{m}\right)$$

(23b) $${\bar{s}}_{r}^{m+1}:=\underset{{\bar{s}}_{r}}{\arg\;\min}L\left({\bar{r}}_{r}^{m+1},{\bar{s}}_{r},u^{m},v^{m},w^{m}\right)$$

(23c) $$u^{m+1}:=u^{m}+A_{r}^{T}{\bar{r}}_{r}^{m+1}+A_{r}^{T}{\bar{s}}_{r}^{m+1}-2x_{r}$$

(23d) $$v^{m+1}:=v^{m}+T\left({\bar{r}}_{r}^{m+1}\right){\bar{s}}_{r}^{m+1}-1$$

(23e) $$w^{m+1}:=w^{m}+{\bar{r}}_{r}^{m+1}-{\bar{s}}_{r}^{m+1}.$$

As can be seen from Equation (23), the updates Equations (23c)–(23e) are straightforward. The subequations Equations (23a) and (23b) are convex and give closed-form solutions. The details of the updates of variables ${\bar{r}}_{r}$ and ${\bar{s}}_{r}$ are presented next.

### 4.1. Update of $\;{\bar{r}}_{r}$

To obtain the (m+1)th update of ${\bar{r}}_{r}$, we take the gradient of Equation (23a) with respect to ${\bar{r}}_{r}$ and equate the result to 0, i.e.,

(24) $$\nabla_{{\bar{r}}_{r}}L\left({\bar{r}}_{r},{\bar{s}}_{r}^{m},u^{m},v^{m},w^{m}\right)=0.$$

The solution to Equation (24) is given by

(25) $${\bar{r}}_{r}^{m+1}=\Xi_{1}^{-1}\xi_{1}$$

where

(26) $$\Xi_{1}=\rho_{1}A_{r}\left(\Theta \right)A_{r}^{T}\left(\Theta \right)+\rho_{2}T^{T}\left({\bar{s}}_{r}\right)T\left({\bar{s}}_{r}\right)+\rho_{3}I$$

and

(27) $$\begin{array}{cc} \xi_{1} & =\rho_{1}A_{r}\left(\Theta \right)\left(2x_{r}-u-A_{r}^{T}\left(\Theta \right){\bar{s}}_{r}\right)\\ \; & \rho_{2}T^{T}\left({\bar{s}}_{r}\right)\left(1-v\right)\rho_{3}\left({\bar{s}}_{r}-w\right)\\ \; & -A_{r}\left(\tilde{\Theta }\right)A_{r}^{T}\left(\tilde{\Theta }\right){\bar{s}}_{r}. \end{array}$$

### 4.2. Update of $\;{\bar{s}}_{r}$

Similar to the ${\bar{r}}_{r}$ update, in the ${\bar{s}}_{r}$ update we take the gradient of Equation (23b) with respect to ${\bar{s}}_{r}$ and equate the result to 0, i.e.,

(28) $$\nabla_{{\bar{s}}_{r}}L\left({\bar{r}}_{r}^{m+1},{\bar{s}}_{r},u^{m},v^{m},w^{m}\right)=0.$$

The solution to Equation (28) is given by

(29) $${\bar{s_{r}}}^{m+1}=\Xi_{2}^{-1}\xi_{2}$$

where

(30) $$\Xi_{2}=\rho_{1}A_{r}\left(\Theta \right)A_{r}^{T}\left(\Theta \right)+\rho_{2}T^{T}\left({\bar{r}}_{r}\right)T\left({\bar{r}}_{r}\right)+\rho_{3}I$$

and

(31) $$\begin{array}{cc} \xi_{2} & =\rho_{1}A_{r}\left(\Theta \right)\left(2x_{r}-u-A_{r}^{T}\left(\Theta \right){\bar{r}}_{r}\right)\\ \; & +\rho_{2}T^{T}\left({\bar{r}}_{r}\right)\left(1-v\right)+\rho_{3}\left({\bar{r}}_{r}+w\right)\\ \; & -A_{r}\left(\tilde{\Theta }\right)A_{r}^{T}\left(\tilde{\Theta }\right){\bar{r}}_{r}. \end{array}$$

### 4.3. Termination Criteria of the Algorithm

Let the primal residuals at iteration m+1 be defined as

(32a) $$d_{pr1}^{m+1}=A_{r}^{T}r_{r}^{m+1}+A_{r}^{T}s_{r}^{m+1}-2x_{r}$$

(32b) $$d_{pr2}^{m+1}=T\left(r_{r}^{m+1}\right)s_{r}^{m+1}-1$$

(32c) $$d_{pr3}^{m+1}=r_{r}^{m+1}-s_{r}^{m+1}$$

and the dual residuals be defined as

(33a) $$d_{rs1}^{m+1}=r_{r}^{m+1}-r_{r}^{m}$$

(33b) $$d_{rs2}^{m+1}=s_{r}^{m+1}-s_{r}^{m}.$$

Then, as suggested by [54], reasonable termination criteria are

(34a) $$\|d_{pr1}^{m+1}{\|}_{2}^{2}\;\leq \;\epsilon_{1}^{pri},$$

(34b) $$\|d_{pr2}^{m+1}{\|}_{2}^{2}\;\leq \;\epsilon_{2}^{pri},$$

(34c) $$\|d_{pr3}^{m+1}{\|}_{2}^{2}\;\leq \;\epsilon_{3}^{pri},$$

(34d) $$\|d_{dr1}^{m+1}{\|}_{2}^{2}\;\leq \;\epsilon^{dual},$$

(34e) $$\|d_{dr2}^{m+1}{\|}_{2}^{2}\;\leq \;\epsilon^{dual}.$$

where $\epsilon_{1}^{pri}$, $\epsilon_{2}^{pri}$, $\epsilon_{3}^{pri}$ are the tolerances for the primal residual and $\epsilon_{1}^{dual}$ is the tolerance for dual residuals. These tolerances, in accordance with [54], are defined as

(35a) $$\epsilon_{1}^{pri}=\sqrt{4N}\epsilon^{abs}+\epsilon^{rel}\max\left\{\|A_{r}^{T}r_{r}^{m+1}{\|}_{2},\|A_{r}^{T}s_{r}^{m+1}{\|}_{2},{\left\|2x_{r}\right\|}_{2}\right\}$$

(35b) $$\epsilon_{2}^{pri}=\sqrt{MN}\epsilon^{abs}+\epsilon^{rel}\max\left\{\|T\left(r_{r}^{m+1}\right)s_{r}^{m+1}{\|}_{2},{\left\|1\right\|}_{2}\right\}$$

(35c) $$\epsilon_{3}^{pri}=\sqrt{2MN}\epsilon^{abs}+\epsilon^{rel}\max\left\{\|r_{r}^{m+1}{\|}_{2},{\left\|s_{r}^{m+1}\right\|}_{2}\right\}$$

(35d) $$\epsilon^{dual}=\sqrt{2MN}\epsilon^{abs}+\epsilon^{rel}{\left\|\rho_{1}w\right\|}_{2}$$

Algorithm 1 summarizes the steps of the algorithm.   
**Algorithm 1:** Summary of the proposed algorithm**Input:**Step (1) **Initialize:** $r_{r}^{0},s_{r}^{0},u^{0},v^{0},w^{0},\rho_{1},\rho_{2},\rho_{3},$ and$\epsilon_{1}^{pri}$, $\epsilon_{2}^{pri}$, $\epsilon_{2}^{pri}$, $\epsilon^{dual}$, m=1.Step (2) **While** the termination criteria, Equation (34), are not satisfied, doStep (3) Update $r_{r}^{m+1}$ using Equation (25)Step (4) Update $s_{r}^{m+1}$ using Equation (29)Step (5) Update $u^{m+1}$ using Equation (23c)Step (6) Update $v^{m+1}$ using Equation (23d)Step (7) Update $w^{m+1}$ using Equation (23e)Step (8) m=m+1Step (9) **End while****Output:**

For clarity, a list of symbols, and their dimensions and descriptions, is provided in Table 2.

### 4.4. Penalty Parameter Selection

Choosing the penalty parameters properly is very important in ADMM. The values of penalty parameters are decreased or increased depending on the values of some predefined tolerances. Different methods can be used choose the penalty parameters, such as hit-and-trial, etc. Another method is to relate the values of the penalty parameters to iteration numbers so that the values of penalty parameters increase or decrease (from the initially defined value) in steps. One standard method is to relate the values of the residual norms with the tolerances by using the concept of ‘residual balancing’ as given by Equation (36).

(36) $$\rho_{k+1}=\left\{\begin{array}{ccc} \eta \rho_{k} & \mathrm{if} & d_{pr1}^{m+1}>\mu \epsilon_{1}^{pri}\\ \rho_{k}/\eta & \mathrm{if} & \epsilon_{1}^{pri}>\mu d_{pr1}^{m+1}\\ \rho_{k} & otherwise \end{array}\right.$$

where $\rho_{k}$ is the penalty parameter, and μ>1 and η>1 are constants, $d_{pr1}^{m+1}$ is the primary residual, and $\epsilon_{1}^{pri}$ is the tolerance.

## 5. Simulation Results and Analysis

In this section, the performance analysis of the algorithm is discussed and the results of some numerical examples are presented to evaluate the performance of the proposed waveform design method. A ULA consisting of M=32 antenna elements having half-wavelength enter-element spacing has been considered at the transmitter and receiver sides. The radar target is located at $\theta_{R}=0^{\circ }$ and the communication user at $\theta_{C}=45^{\circ }$. The desired radar waveform is based on linear frequency modulation (LFM). Similarly, the desired communication waveform uses the QPSK modulation scheme. We have considered $N_{s}=1$ symbols and $N_{b}=2$ bits per symbol. Thus, each waveform carries 2 bits of information per pulse repetition interval (PRI).

Different experiments are performed to evaluate the performance in different scenarios. Because both radar and communication receivers expect some desired waveforms, coherent detection can be used to match the received signal waveform with the desired waveform. Monte Carlo simulations are conducted to evaluate the performance of communication for different values of the signal-to-noise ratio (SNR).

The proposed method is compared with the far-field radiated emission design (FFRED) [56], the iterative optimization technique (using directly normalized waveforms) [26], and the theoretical values. In the FFRED method, 0%, 10%, and 40% of the total power is allocated to the orthogonal complement waveform, of which the FFRED-40% has the best performance. The authors of [26] proposed several waveform design methods. One method designed non-constant modulus waveforms and had a closed-form solution to the waveform design problem. They also proposed an iterative method for constant modulus waveforms. However, being computationally complex, they used the results of the first method using non-constant modulus waveforms and used iterative optimization for further refining those waveforms. They defined this method as ‘directly normalized’ in their simulations.

### 5.1. Computational Complexity Analysis

The computational complexity analysis is used to estimate the amount of computational resources (such as time and memory) required to run an algorithm. It provides an understanding of how the algorithm scales with the input size and helps in optimizing the algorithm or selecting alternative approaches if the complexity is too high.

To calculate the computational complexity of the proposed algorithm, each part of the code is analyzed to determine the number of operations or iterations performed in terms of the input size. In the code, the main loop iterates ‘iter’ number of times. Within each iteration, there are multiple calculations and operations performed, such as matrix multiplications, norm calculations, and updates of variables. The complexity of each of these operations is analyzed and sums them up to obtain an overall complexity estimate for the code. In addition, the input size-dependent variables, such as M and L, and their impact on the complexity are also considered.

The proposed algorithm has high computational complexity as it is cubic in nature. This is because it involves a matrix inversion operation. After the matrix inversion operation, the other main time-consuming operations are matrix multiplication operations.

For r updates, the calculation of $\Xi_{1}$ takes $O(KM^{2}L^{2})$ and the calculation of γ takes $O(M^{2}L^{2})$; therefore, the complexity of the update of r using Equation (25) is $O(KM^{2}L^{2}+M^{2}L^{2}+M^{3}L^{3})$. Overall, the computational complexity of the algorithm is $O(2\left(KM^{2}L^{2}+M^{2}L^{2}+M^{3}L^{3}\right))$ at each iteration.

### 5.2. Data Rate Performance

The communication data rate is

(37) $$R=N_{b}\times N_{s}\times f_{PRF},$$

where $N_{b}$ is the number of bits per symbol, $N_{s}$ is the number of symbols in one pulse, and $f_{PRF}$ is the pulse repetition frequency.

### 5.3. ADMM Convergence Analysis

**Figure 2:**
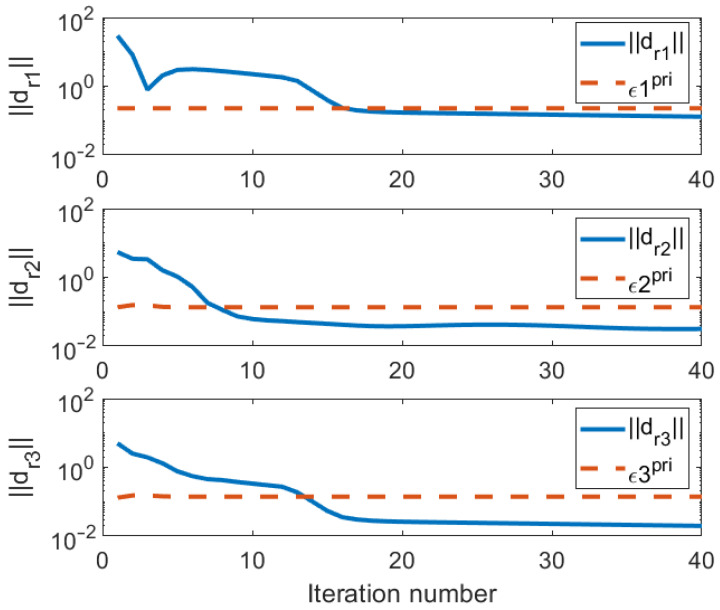
Norms of primary residuals per iteration.

**Figure 3:**
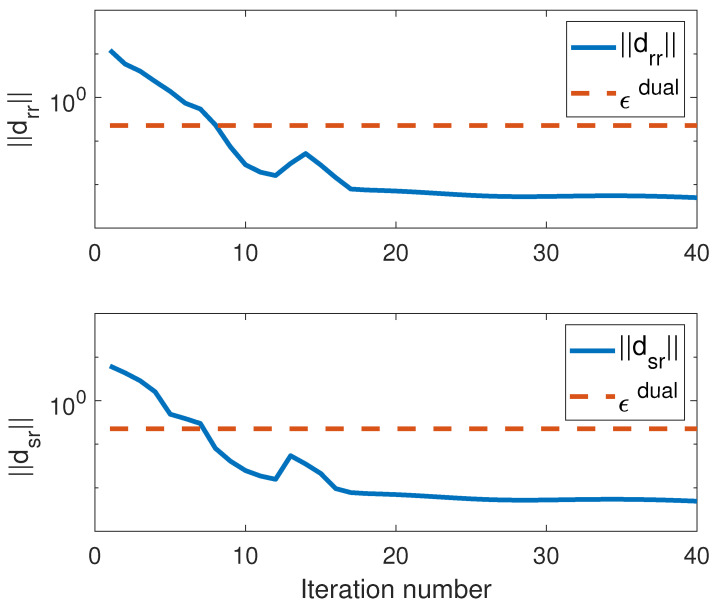
Norms of dual residuals per iteration.

Plots of the norms of the primal and dual residuals, $d_{pr1}$, $d_{pr2}$, $d_{pr3}$, $d_{dr2}$, $d_{dr2}$, and the stopping criteria limits $\epsilon_{1}^{pri}$, $\epsilon_{2}^{pri}$, $\epsilon_{2}^{pri}$, $\epsilon^{dual}$ against the iteration numbers are shown in Figure 2 and Figure 3. The plots show that the stopping conditions are met within 20 iterations.

**Figure 4:**
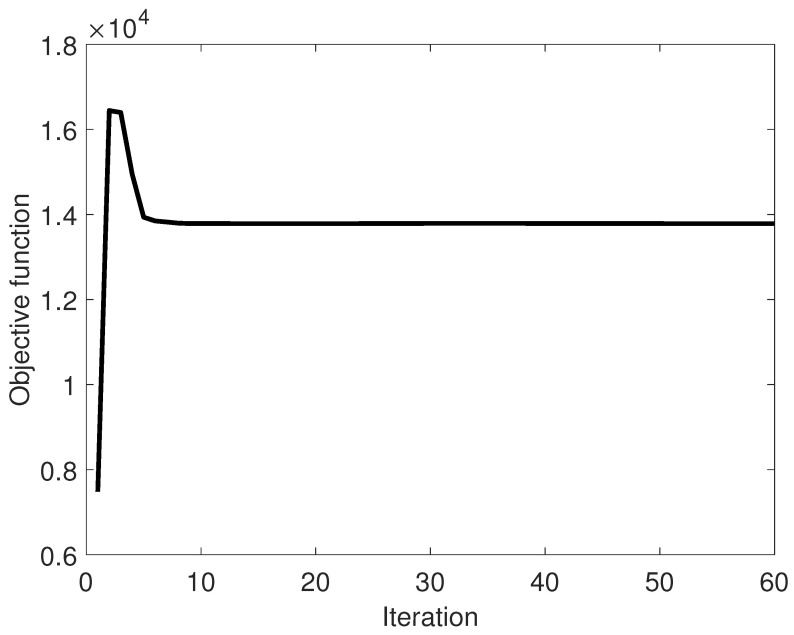
Convergence of the objective function Equation (18).

Figure 4 shows a plot of the objective function values. The objective function is the first line of Equation (18). As is obvious in the figure, the objective function settles within 10 iterations. This is in accordance with the settling of the primary and dual residuals, Figure 2 and Figure 3.

### 5.4. Beampattern Analysis

**Figure 5:**
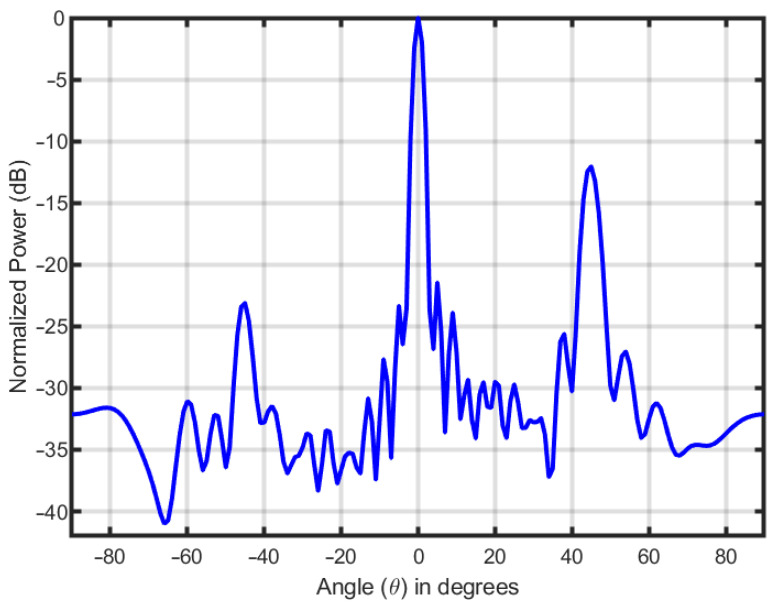
The transmit beampattern formed by the DFRC system with 32 antenna elements.

Figure 5 shows the transmit beampattern formed by the waveform matrix **S** designed through the proposed ADMM-based approach for a DFRC system with 32 antenna elements.

**Figure 6:**
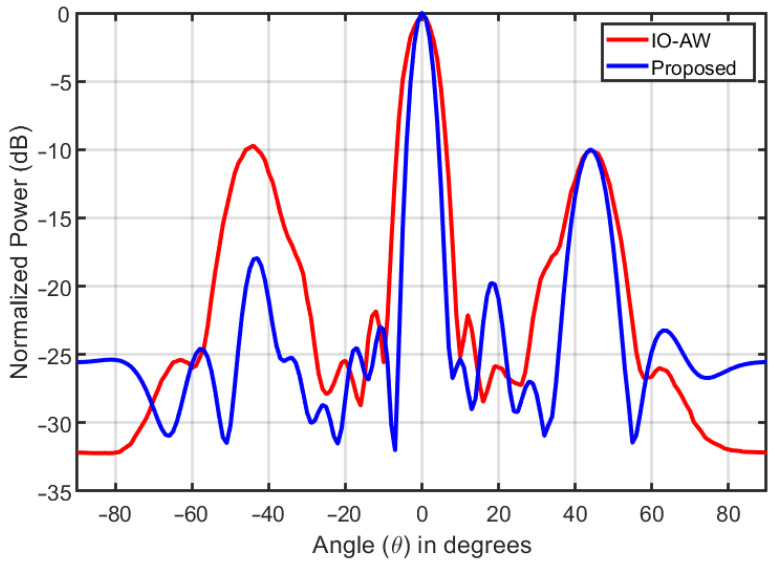
The transmit beampattern formed by the systems.

Figure 6 shows the transmit beampatterns as synthesized by the waveform matrix **S** designed through the proposed ADMM-based approach and that of iterative optimization with amplitude weighting (IO-AW) as reported in [46]. In both cases, the systems have 16 antenna elements, and the radar target is located at $\theta_{R}=0^{\circ }$ and the communication user at $\theta_{C}=45^{\circ }$. Moreover, in both cases, the power of the desired radar waveform is designed to be 10 dB more than that of the communication waveform. As can be seen in the figure, the IO-AW method leaks power at $-45^{{}^{\circ}}$ or, in other words, makes a mirror lobe toward a direction where there is no communication user. Otherwise, the sidelobe levels of the two beampatterns are almost the same. Thus, the beampattern formed through the proposed method outperforms the beampattern formed through IO-AW.

### 5.5. Waveform Error Analysis

Normalized waveform error is a performance metric that can be used to describe how closely the desired waveforms match the synthesized waveforms. Moreover, it can also give convergence performance, as the faster it decreases with the increasing number of iterations, the better the performance of the algorithm. Mathematically, this metric, denoted here as $\eta_{i}$, is defined as

(38) $$\eta_{i}=\frac{{∥X_{i}∥}_{F}^{2}}{{∥X∥}_{F}^{2}}$$

where

(39) $$X_{i}=A^{H}\left(\Theta \right)S_{i}.$$

**Figure 7:**
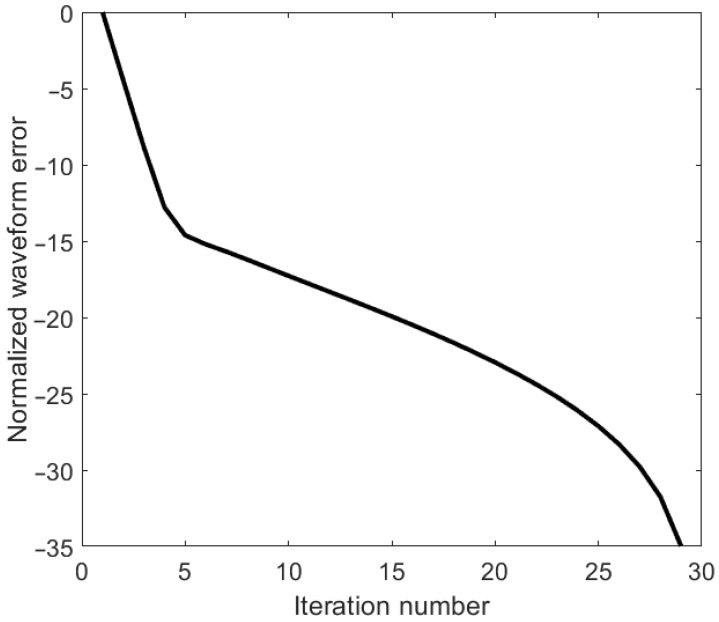
Normalized waveform error.

Figure 7 shows the normalized waveform error plotted against the iterations.

Table 3 provides a comparison of different methods for the normalized waveform error.

**Figure 8:**
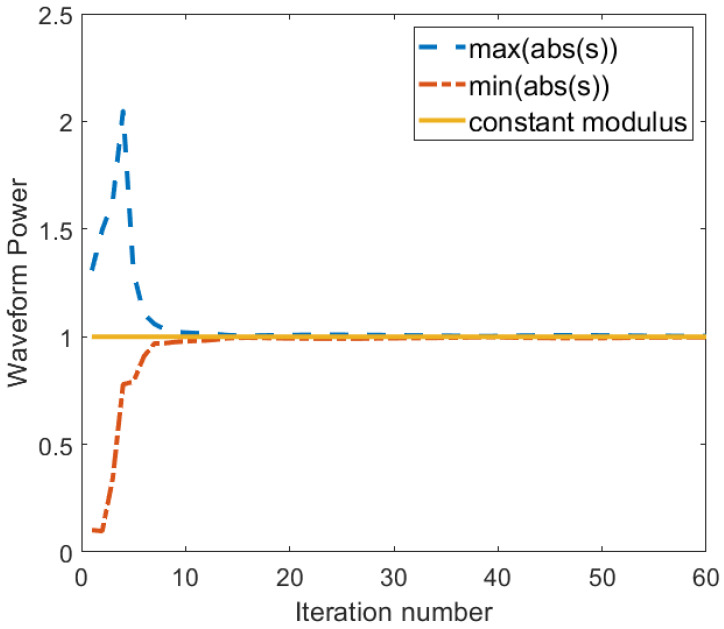
The waveform modulus per iteration showing the constant modulus constraint is satisfied.

Figure 8 shows how the CM constraint is met. The plots in the figure show the maximum and the minimum modulus samples of S, and the desired uni-modulus samples, at each iteration. As can be seen in the plots, the maximum and the minimum values settle at 1 at about the 10th iteration.

### 5.6. Radar Performance Analysis

The radar performance evaluation is provided in two figures: the first figure compares the desired LFM waveform for radar and the far-field synthesized waveform, whereas the second gives the detection probability (pD) versus the SNR.

**Figure 9:**
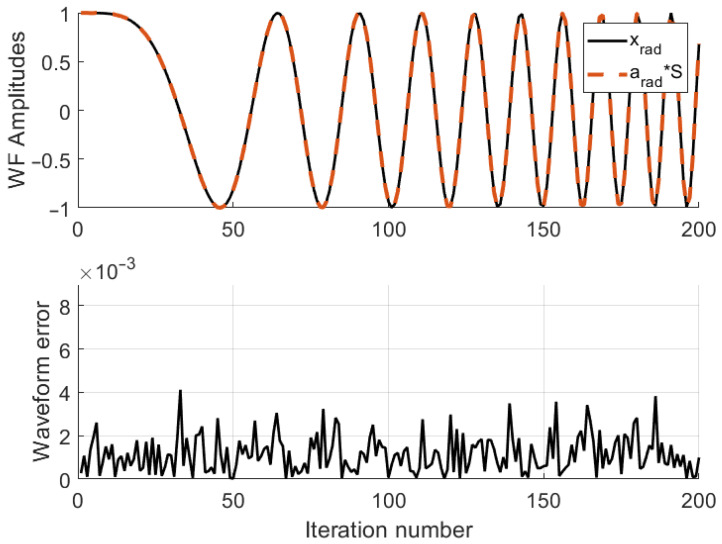
Synthesized radar waveform: (**Upper**) desired waveform vs. far-field synthesized waveform, (**Lower**) difference between the desired waveform vs. far-field synthesized waveform. The asterisk sign (*) represents vector-matrix multiplication.

The waveform synthesized in the radar direction is shown in Figure 9. As shown in Figure 9 (upper), the synthesized radar waveform and the desired radar waveform seem almost identical, which validates the efficiency of the proposed scheme. However, there are small differences between the waveforms, shown in Figure 9 (lower). These differences or sample errors are defined as

(40) $$e_{R}=x_{R}-a\left(\theta_{R}\right)S.$$

**Figure 10:**
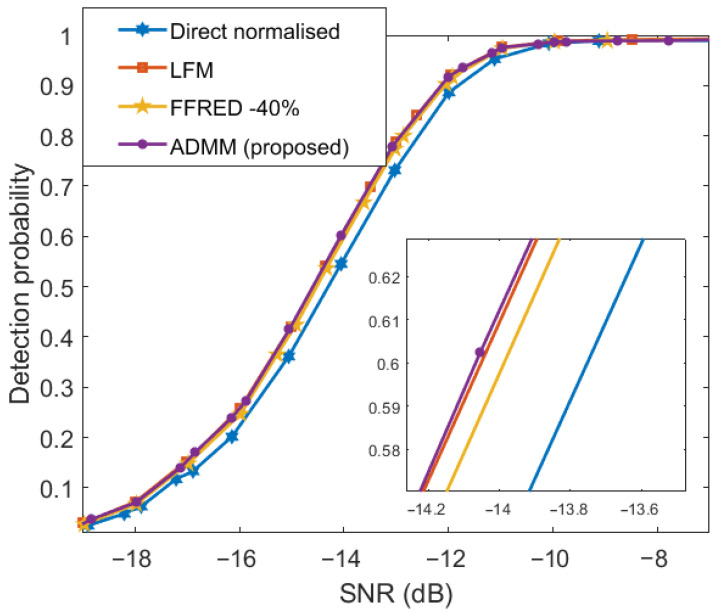
Comparison of different methods: detection probability vs. SNR.

Figure 10 shows the graph of detection probability plotted against the SNR. The probability of a false alarm is set as $10^{-4}$. For comparison, the pD versus SNR graphs of other methods are also provided. As the figure shows, FFRED-40% [56] has the best pD, which is almost the same as that of the desired LFM. However, the graph of the proposed method is so close that the difference becomes visible upon zooming in on the plots. At the same time, the proposed method provides better pD than that of the directly normalized method [26].

### 5.7. Communication Performance Analysis

Like radar performance, communication performance, too, is evaluated by two figures: the first figure gives a comparison of the desired communication waveform and the far-field synthesized waveform, whereas the second figure gives the SER versus the SNR.

**Figure 11:**
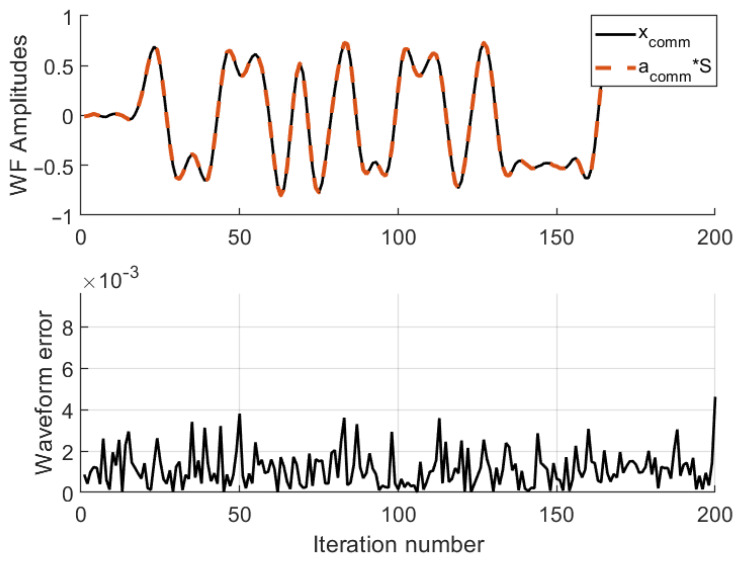
Synthesized communication waveform: (**Upper**) desired waveform vs. far-field synthesized waveform, (**lower**) difference between the desired waveform vs. far-field synthesized waveform.

The waveform synthesized in the communication direction is shown in Figure 11. As with radar waveforms, the synthesized and the desired communication waveforms seem almost exact. The sample errors in this case, Figure 11 (lower), are defined as

(41) $$e_{C}=x_{C}-a\left(\theta_{C}\right)S.$$

**Figure 12:**
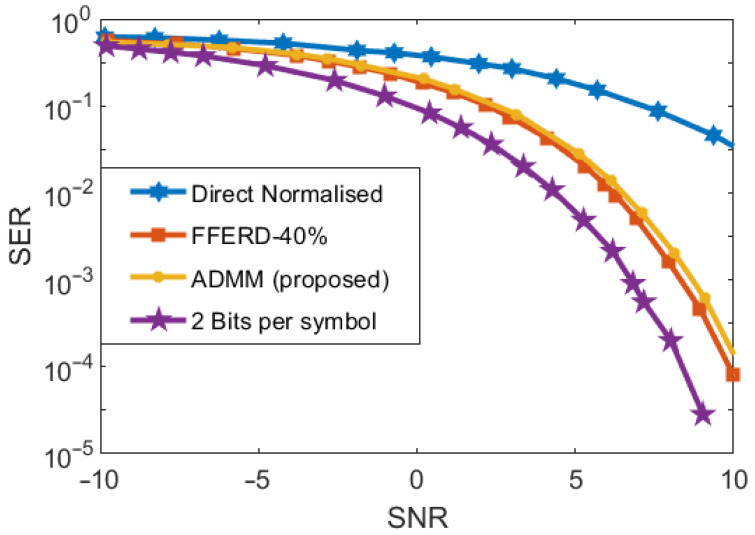
Comparison of different methods: SER vs. SNR.

Figure 12 shows the SER plotted against the SNR. Again, for comparison, the SER versus SNR graphs of other methods are provided. The ‘2 bits per symbol’ graph represents the theoretical values. Again, the graphs of FFRED-40% [56] and the proposed ADMM method are very close, although FFRED-40% has a relatively better performance. Both methods outperform the directly normalized waveform method [26].

## 6. Conclusions

A method for designing the constant modulus waveforms for MIMO dual-function radar-communication systems was proposed in this paper. The design problem was mathematically formulated as an optimization problem subject to the constraints of waveform synthesis and constant modulus. The optimization problem thus formulated, being non-convex and NP-hard, was solved iteratively using an ADMM framework. Importantly, the designed waveforms approximated a desired beampattern in terms of a high-gain radar beam and a slightly high gain communication beam while maintaining a desired low sidelobe level. The designed waveforms ensured an improved detection probability and an improved bit error rate (BER) for the radar and communications parts, respectively. Based on the simulation results, the effectiveness of the proposed scheme has been validated.

## Figures and Tables

**Table 1:** List of abbreviations and their full forms.

Abbreviation	Description
ADMM	Alternating Direction Method of Multipliers
ALM	Augmented Lagrangian Method
ATC	Air Traffic Control
BER	Bit Error Rate
CM	Constant Modulus
CMC	Constant Modulus Constraint
CRSS	Communication-Radar Spectrum Sharing
DFRC	Dual-Function Radar Communication
EW	Electronic Warfare
FANET	Flying Ad hoc Network
FFRED	Far-Field Radiated Emission Design
GA	Genetic Algorithm
IO-AW	Iterative Optimization with Amplitude Weighting
IRCS	Integrated Radar and Communication System
ISAC	Integrated Sensing and Communication
LFM	Linear Frequency Modulation
MIMO	Multi-Input Multi-Output
OFDM	Orthogonal Frequency Division Multiplex
PAPR	Peak-to-Average Power Ratio
PRI	Pulse Repetition Interval
QPSK	Quadrature Phase-Shift Keying
RCC	Radar-Communication Coexistence
SER	Symbol Error Rate
ULA	Uniform Linear Array
WS	Waveform Synthesis

**Table 2:** List of symbols.

Symbol	Dimension	Description
M	1×1	Number of antennas
N	1×1	Number of samples
K	1×1	Number of sidelobes
d	1×1	Antenna inter-element spacing
$I_{N}$	N×N	Identity matrix
λ	1×1	Wavelength
$s_{m}\left(n\right)$	1×1	*n*th sample of a discrete waveform
s(n)	M×1	*n*th samples of the waveforms transmitted by all antennas
S	M×N	Space-time transmit waveform matrix
$\bar{s}$	MN×1	Vector version of s
${\bar{s}}_{r}$	2MN×1	Real-valued version of $\bar{s}$
$x_{R}$	N×1	Desired radar waveform
$x_{C}$	N×1	Desired communication waveform
X	2×N	Combination of desired communication waveform as a matrix
$\bar{x}$	2N×1	Vector version of X
${\bar{x}}_{r}$	4N×1	Real-valued version of $\bar{x}$
$a(\theta_{R})$	M×1	Steering vector in radar direction
$a(\theta_{C})$	M×1	Steering vector in communication direction
$A\left(\Theta \right)$	M×2	Combination of $a(\theta_{R}$ and $a(\theta_{C}$
$\bar{A}\left(\Theta \right)$	MN×2N	Vector version of $A\left(\Theta \right)$
${\bar{A}}_{r}\left(\Theta \right)$	2MN×4N	Real-valued version of $\bar{A}\left(\Theta \right)$
$A\left(\tilde{\Theta }\right)$	M×K	Combination of sidelobe steering vectors
$\bar{A}\left(\tilde{\Theta }\right)$	MN×KN	Vector version of $A\left(\tilde{\Theta }\right)$
${\bar{A}}_{r}\left(\tilde{\Theta }\right)$	2MN×2KN	Real-valued version of $\bar{A}\left(\tilde{\Theta }\right)$
**u**	4N×1	Dual variable
**v**	MN×1	Dual variable
**w**	2MN×1	Dual variable
η, μ	1×1	Positive constants
$\rho_{1}$, $\rho_{2}$, $\rho_{3}$	1×1	Penalty parameters

**Table 3:** Comparison of different methods for normalized waveform error.

Method	Waveform Modulus	Normalized Waveform Error/dB
FFRED-0%	Non-constant	−320.08
FFRED-10%	Constant	−34.08
FFRED-40%	Constant	−113.56
MNO	Non-constant	−312.06
IO	Constant	−39.40
IO-AW	Constant	−40.90
ADMM-based (Proposed)	Constant	−35

## Data Availability

Not applicable.

## References

[1] Benedetto F., Mastroeni L., Quaresima G. Auction-based Theory for Dynamic Spectrum Access: A Review. Proceedings of the 2021 44th International Conference on Telecommunications and Signal Processing (TSP).

[2] Chapin J., Lehr W. (2011). Mobile Broadband Growth, Spectrum Scarcity, and Sustainable Competition.

[3] Noam E. (1998). Spectrum auctions: Yesterday’s heresy, today’s orthodoxy, tomorrow’s anachronism. Taking the next step to open spectrum access. J. Law Econ..

[4] Martínez-Santos F., Frias Z., Escribano Á. (2022). What drives spectrum prices in multi-band spectrum markets? An empirical analysis of 4G and 5G auctions in Europe. Appl. Econ..

[5] Sridhar V., Prasad R. (2021). Analysis of spectrum pricing for commercial mobile services: A cross country study. Telecommun. Policy.

[6] Cave M. (2021). The Past, Present and Future of Spectrum Auctions. The Debates Shaping Spectrum Policy.

[7] Munir M.F., Basit A., Khan W., Saleem A., Al-salehi A. (2022). A Comprehensive Study of Past, Present, and Future of Spectrum Sharing and Information Embedding Techniques in Joint Wireless Communication and Radar Systems. Wirel. Commun. Mob. Comput..

[8] Papadias C.B., Ratnarajah T., Slock D.T. (2020). Spectrum Sharing: The Next Frontier in Wireless Networks.

[9] Cheema A.A., Salous S. (2019). Spectrum occupancy measurements and analysis in 2.4 GHz WLAN. Electronics.

[10] Cheema A.A., Salous S. (2016). Digital FMCW for ultrawideband spectrum sensing. Radio Sci..

[11] Peha J.M. (2005). Approaches to spectrum sharing. IEEE Commun. Mag..

[12] Mir S., Bari I., Kamal M., Ali H. (2021). Constraint waveform design for spectrum sharing under coexistence of radar and communication systems. IEEE Access.

[13] Huang Y., Hu S., Ma S., Liu Z., Xiao M. (2022). Designing Low-PAPR Waveform for OFDM-based RadCom Systems. IEEE Trans. Wirel. Commun..

[14] Liu F., Cui Y., Masouros C., Xu J., Han T.X., Eldar Y.C., Buzzi S. (2022). Integrated sensing and communications: Towards dual-functional wireless networks for 6G and beyond. IEEE J. Sel. Areas Commun..

[15] Cheng X., Duan D., Gao S., Yang L. (2022). Integrated Sensing and Communications (ISAC) for Vehicular Communication Networks (VCN). IEEE Internet Things J..

[16] Zheng L., Lops M., Eldar Y.C., Wang X. (2019). Radar and communication coexistence: An overview: A review of recent methods. IEEE Signal Process. Mag..

[17] Tavik G.C., Hilterbrick C.L., Evins J.B., Alter J.J., Crnkovich J.G., de Graaf J.W., Habicht W., Hrin G.P., Lessin S.A., Wu D.C. (2005). The advanced multifunction RF concept. IEEE Trans. Microw. Theory Tech..

[18] Moghaddasi J., Wu K. (2016). Multifunctional transceiver for future radar sensing and radio communicating data-fusion platform. IEEE Access.

[19] Hassanien A., Amin M.G., Aboutanios E., Himed B. (2019). Dual-function radar communication systems: A solution to the spectrum congestion problem. IEEE Signal Process. Mag..

[20] Tsinos C.G., Arora A., Chatzinotas S., Ottersten B. (2021). Joint transmit waveform and receive filter design for dual-function radar-communication systems. IEEE J. Sel. Top. Signal Process..

[21] Wen C., Huang Y., Davidson T.N. (2023). Efficient Transceiver Design for MIMO Dual-Function Radar-Communication Systems. IEEE Trans. Signal Process..

[22] Rong J., Liu F., Miao Y. (2022). Integrated Radar and Communications Waveform Design Based on Multi-Symbol OFDM. Remote Sens..

[23] Chiriyath A.R., Paul B., Bliss D.W. (2017). Radar-communications convergence: Coexistence, cooperation, and co-design. IEEE Trans. Cogn. Commun. Netw..

[24] Singh R., Saluja D., Kumar S. (2021). R-Comm: A traffic based approach for joint vehicular radar-communication. IEEE Trans. Intell. Veh..

[25] Ma D., Shlezinger N., Huang T., Liu Y., Eldar Y.C. (2020). Joint radar-communication strategies for autonomous vehicles: Combining two key automotive technologies. IEEE Signal Process. Mag..

[26] Han L., Wu K. (2012). 24-GHz integrated radio and radar system capable of time-agile wireless communication and sensing. IEEE Trans. Microw. Theory Tech..

[27] Kang B., Aldayel O., Monga V., Rangaswamy M. (2018). Spatio-spectral radar beampattern design for coexistence with wireless communication systems. IEEE Trans. Aerosp. Electron. Syst..

[28] Sodagari S., Khawar A., Clancy T.C., McGwier R. A projection based approach for radar and telecommunication systems coexistence. Proceedings of the 2012 IEEE Global Communications Conference (GLOBECOM).

[29] Babaei A., Tranter W.H., Bose T. A nullspace-based precoder with subspace expansion for radar/communications coexistence. Proceedings of the 2013 IEEE Global Communications Conference (GLOBECOM).

[30] Mahal J.A., Khawar A., Abdelhadi A., Clancy T.C. (2017). Spectral coexistence of MIMO radar and MIMO cellular system. IEEE Trans. Aerosp. Electron. Syst..

[31] Li B., Petropulu A.P. (2017). Joint transmit designs for coexistence of MIMO wireless communications and sparse sensing radars in clutter. IEEE Trans. Aerosp. Electron. Syst..

[32] Li B., Petropulu A.P., Trappe W. (2016). Optimum co-design for spectrum sharing between matrix completion based MIMO radars and a MIMO communication system. IEEE Trans. Signal Process..

[33] Zhu J., Cui Y., Mu J., Jing X. OFDM-based Dual-Function Radar-Communications: Optimal Resource Allocation for Fairness. Proceedings of the 2022 IEEE 95th Vehicular Technology Conference:(VTC2022-Spring).

[34] Johnston J., Venturino L., Grossi E., Lops M., Wang X. (2022). MIMO OFDM dual-function radar-communication under error rate and beampattern constraints. IEEE J. Sel. Areas Commun..

[35] Ahmed A., Zhang Y.D., Hassanien A. (2021). Joint radar-communications exploiting optimized OFDM waveforms. Remote Sens..

[36] Xu Z., Petropulu A. (2021). A wideband dual function radar communication system with sparse array and ofdm waveforms. arXiv.

[37] Liu Y., Liao G., Chen Y., Xu J., Yin Y. (2020). Super-resolution range and velocity estimations with OFDM integrated radar and communications waveform. IEEE Trans. Veh. Technol..

[38] Liu Y., Liao G., Yang Z. (2019). Robust OFDM integrated radar and communications waveform design based on information theory. Signal Process..

[39] Wen C., Huang Y., Zheng L., Liu W., Davidson T.N. (2023). Transmit Waveform Design for Dual-Function Radar-Communication Systems via Hybrid Linear-Nonlinear Precoding. IEEE Trans. Signal Process..

[40] Nowak M.J., Zhang Z., LoMonte L., Wicks M., Wu Z. (2017). Mixed-modulated linear frequency modulated radar-communications. IET Radar Sonar Navig..

[41] Zhang Y., Li Q., Huang L., Dai K., Song J. Waveform design for joint radar-communication with nonideal power amplifier and outband interference. Proceedings of the 2017 IEEE Wireless Communications and Networking Conference (WCNC).

[42] Hassanien A., Amin M.G., Zhang Y.D., Ahmad F. (2016). Phase-modulation based dual-function radar-communications. IET Radar Sonar Navig..

[43] Hassanien A., Amin M.G., Zhang Y.D., Ahmad F. (2015). Dual-function radar-communications: Information embedding using sidelobe control and waveform diversity. IEEE Trans. Signal Process..

[44] Ahmed A., Zhang Y.D., Gu Y. (2018). Dual-function radar-communications using QAM-based sidelobe modulation. Digit. Signal Process..

[45] Tang B., Stoica P. (2022). MIMO multifunction RF systems: Detection performance and waveform design. IEEE Trans. Signal Process..

[46] Jiang M., Liao G., Yang Z., Liu Y., Chen Y. (2021). Integrated radar and communication waveform design based on a shared array. Signal Process..

[47] Liu X., Huang T., Liu Y., Zhou J. Constant Modulus Waveform Design for Joint Multiuser MIMO Communication and MIMO Radar. Proceedings of the 2021 IEEE Wireless Communications and Networking Conference Workshops (WCNCW).

[48] Mancuso V., Alouf S. (2011). Reducing costs and pollution in cellular networks. IEEE Commun. Mag..

[49] De Maio A., De Nicola S., Huang Y., Luo Z.Q., Zhang S. (2008). Design of phase codes for radar performance optimization with a similarity constraint. IEEE Trans. Signal Process..

[50] Cui G., Li H., Rangaswamy M. (2013). MIMO radar waveform design with constant modulus and similarity constraints. IEEE Trans. Signal Process..

[51] Liu F., Masouros C., Amadori P.V., Sun H. (2017). An efficient manifold algorithm for constructive interference based constant envelope precoding. IEEE Signal Process. Lett..

[52] Wang X., Hassanien A., Amin M.G. (2018). Dual-function MIMO radar communications system design via sparse array optimization. IEEE Trans. Aerosp. Electron. Syst..

[53] Liu F., Zhou L., Masouros C., Li A., Luo W., Petropulu A. (2018). Toward dual-functional radar-communication systems: Optimal waveform design. IEEE Trans. Signal Process..

[54] Boyd S., Parikh N., Chu E., Peleato B., Eckstein J. (2011). Distributed optimization and statistical learning via the alternating direction method of multipliers. Found. Trends® Mach. Learn..

[55] Hong M., Luo Z.Q., Razaviyayn M. (2016). Convergence analysis of alternating direction method of multipliers for a family of nonconvex problems. SIAM J. Optim..

[56] McCormick P., Blunt S., Metcalf J. Simultaneous radar and communications emissions from a common aperture, part I: Theory. Proceedings of the 2017 IEEE Radar Conference (RadarConf).

